# *In vivo* phage display screening for tumor vascular targets in glioblastoma identifies a llama nanobody against dynactin-1-p150^Glued^

**DOI:** 10.18632/oncotarget.12261

**Published:** 2016-09-26

**Authors:** Sanne A.M. van Lith, Ilse Roodink, Joost J.C. Verhoeff, Petri I. Mäkinen, Jari P. Lappalainen, Seppo Ylä-Herttuala, Jos Raats, Erwin van Wijk, Ronald Roepman, Stef J. Letteboer, Kiek Verrijp, William P.J. Leenders

**Affiliations:** ^1^ Department of Pathology, RadboudUMC, 6500 HB, Nijmegen, The Netherlands; ^2^ Department of Radiotherapy, Amsterdam Medical Center, 1100 DD, Amsterdam, The Netherlands; ^3^ Department of Biotechnology and Molecular Medicine, University of Eastern Finland, FI-70211, Kuopio, Finland; ^4^ Science Service Center and Gene Therapy Unit, Kuopio University Hospital, 70210 Kuopio, Finland; ^5^ Modiquest BV, LSP, Molenstraat 110, 5342 CC, Oss, The Netherlands; ^6^ Department of Otorhinolaryngology, RadboudUMC, 6500 HB, Nijmegen, The Netherlands; ^7^ Department of Genetics, RadboudUMC, 6500 HB, Nijmegen, The Netherlands

**Keywords:** glioma, stroma, targeting, nanobody, macrophages

## Abstract

Diffuse gliomas are primary brain cancers that are characterised by infiltrative growth. Whereas high-grade glioma characteristically presents with perinecrotic neovascularisation, large tumor areas thrive on pre-existent vasculature as well. Clinical studies have revealed that pharmacological inhibition of the angiogenic process does not improve survival of glioblastoma patients. Direct targeting of tumor vessels may however still be an interesting therapeutic approach as it allows pinching off the blood supply to tumor cells. Such tumor vessel targeting requires the identification of tumor-specific vascular targeting agents (TVTAs).

Here we describe a novel TVTA, C-C7, which we identified via *in vivo* biopanning of a llama nanobody phage display library in an orthotopic mouse model of diffuse glioma. We show that C-C7 recognizes a subpopulation of tumor blood vessels in glioma xenografts and clinical glioma samples. Additionally, C-C7 recognizes macrophages and activated endothelial cells in atherosclerotic lesions. By using C-C7 as bait in yeast-2-hybrid (Y2H) screens we identified dynactin-1-p150Glued as its binding partner. The interaction was confirmed by co-immunostainings with C-C7 and a commercial anti-dynactin-1-p150Glued antibody, and via co-immunoprecipitation/western blot studies. Normal brain vessels do not express dynactin-1-p150^Glued^ and its expression is reduced under anti-VEGF therapy, suggesting that dynactin-1-p150^Glued^ is a marker for activated endothelial cells.

In conclusion, we show that *in vivo* phage display combined with Y2H screenings provides a powerful approach to identify tumor-targeting nanobodies and their binding partners. Using this combination of methods we identify dynactin-1-p150^Glued^ as a novel targetable protein on activated endothelial cells and macrophages.

## INTRODUCTION

In order to grow and disseminate, tumors depend on an adequate blood supply [1, 2]. Based on the assumption that tumors arrange their own blood supply via induction of angiogenesis, in the last decade angiogenesis inhibitors (e.g. the anti-VEGF-A antibody bevacizumab or VEGFR2-specific tyrosine kinase inhibitors) have been widely implemented in clinical practice for a number of tumor types [3, 4]. Effects of anti-angiogenesis (mostly in combination with chemotherapy) are however mostly transient [5, 6] and cancer patients who undergo anti-angiogenic treatment mostly experience recurrences, one possible cause being that tumors adopt an invasive phenotype to accommodate their metabolic needs [7–10]. Resistance to anti-angiogenic treatment may also be related to heterogeneity of the tumor vasculature. Vessel formation in cancer is a multistep process and consequently cancers contain neovessels in different stages of development [11, 12]. Because endothelial cells in matured vessels are less dependent on VEGF-VEGFR2 signalling, these are also more refractory to VEGF inhibition [13].

Vessel heterogeneity is especially prominent in glioblastoma, a highly malignant brain tumor of glial origin [14]. One hallmark of glioblastoma is the presence of large areas of diffuse infiltrative growth in which tumor cells thrive on pre-existent vessels (co-option) without the need for angiogenesis [15, 16]. Another characteristic of glioblastoma is focal angiogenesis in the vicinity of areas of necrosis, and this has been rationale for preclinical and clinical testing of anti-VEGF therapy for recurrent and primary diagnosed glioblastoma. Such studies have shown effective inhibition of angiogenesis, however without preventing tumor progression in areas of diffuse infiltrative growth [17–19]. This growth pattern thus provides tumors with a route of escape from anti-angiogenic therapies [5, 20, 21] and recent clinical trials confirmed a lack of prolonged survival of glioblastoma patients upon treatment with bevacizumab [22, 23].

An alternative approach to deprive cancers from blood is direct anti-vascular therapy that aims at tumor-specific thrombosis and infarction. Such an approach requires that tumor vascular targeting agents (TVTAs) are developed with high enough specificity for tumor vasculature. Available TVTAs (e.g. RGD peptides or cilengitide, targeting endothelial αvβ3 integrin [24, 25], the L19 single chain antibody that targets the ED-B fragment of fibronectin [26], anti-V-CAM antibodies [27], anti-plexinD1 antibodies [28]) are directed against newly formed vessels and do not target tumor vessels in more matured stages of development.

Biopanning of peptide or single chain-antibody phage display libraries is a powerful technique that allows identification and isolation of tumor endothelium binding partners [29–32]. Nanobodies^®^ (*V*ariable *Heavy* chain domains of *Heavy* chain antibodies or VHHs) are recombinant antibodies, cloned from cameloid IgG2 and IgG3 heavy chain-only antibodies (V-H) and consist of a single polypeptide chain, making this class of antibodies suitable for display on phages without significant loss of affinity [33, 34]. Their small size (15–18 kDa) and stability make nanobodies to an attractive class of diagnostic and therapeutic compounds [35]. Nanobodies against epidermal growth factor receptor (EGFR) or carcinoembryonic antigen (CEA) have already successfully been used for *in vivo* diagnosis and therapy [36–38].

In a search for novel relevant nanobody-based TVTAs we performed *in vivo* biopannings with a nanobody phage display library [29]. As a tumor model we utilized mice carrying orthotopic E98 human glioma xenografts that characteristically display both angiogenesis-dependent growth and diffuse infiltrative growth [18, 39]. We identified a novel nanobody, C-C7, that targets a subpopulation of tumor blood vessels. Using C-C7 as a bait protein in yeast-2-hybrid screens we identified dynactin-1-p150^Glued^ as its binding partner.

## RESULTS

### *In vivo* selection of tumor vessel binding phages in cerebral E98 xenografts

**Figure 1:**
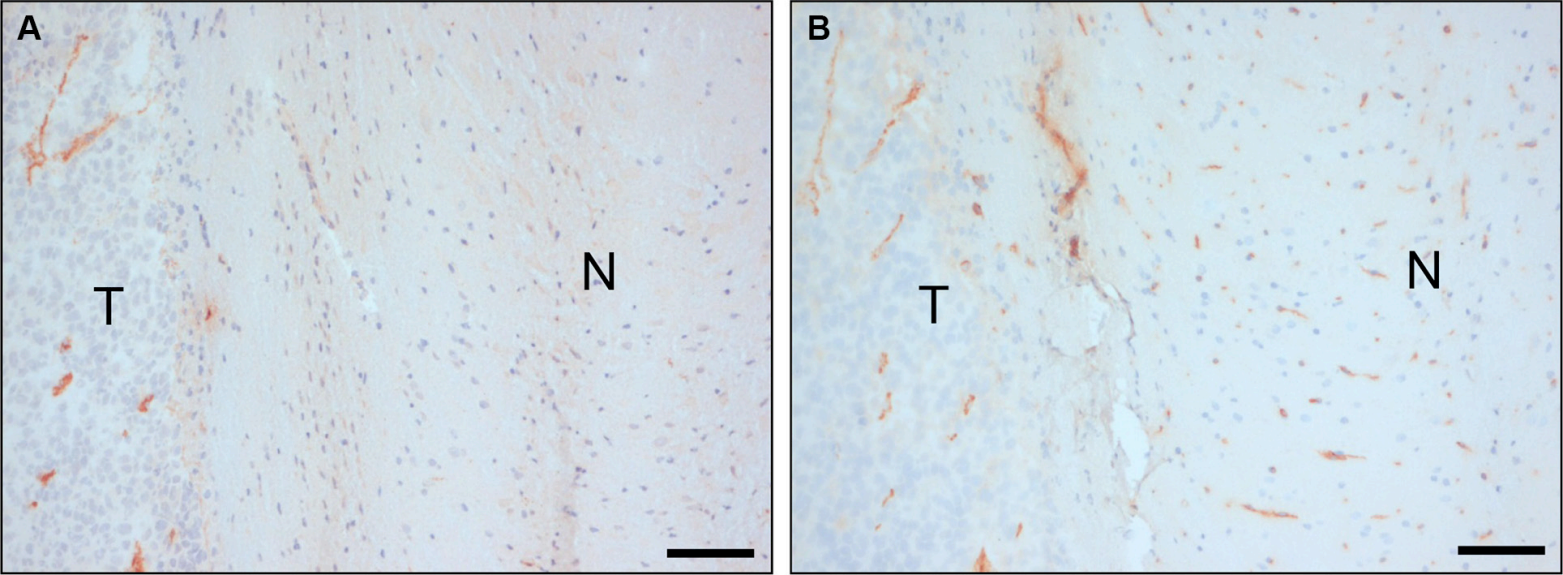
*In vivo* biopanning of a Llama phage library in an animal model of orthotopic glioma Anti-M13-p8 (**A**) and anti-CD34 (**B**) immunostainings of sections of E98 xenografts in mouse brain after intravenous injection of 10^12^ phages of the nanobody-phage display library, and cardiac perfusion. Note that phages are associated with tumor vasculature, but to a lesser extent with blood vessels in normal brain. N = normal, T = tumor. Bars correspond to 100 μm.

A nanobody-displaying phage library [28] was intravenously injected in mice carrying intracerebral E98 xenografts and irrelevant phages were removed from the circulation by cardiac perfusion. We chose to use mice carrying orthotopic E98 xenografts because these tumors display both areas of angiogenesis and diffuse infiltrative growth [39]. Similarly to our previous work using different tumor xenograft models and other phage libraries [29], anti-M13 immunostainings demonstrated already a tumor-specific vessel localization of phages after the first round of biopanning (Figure 1, compare the anti-M13 immunostaining in panel A to the endothelial cell CD34 staining in panel B). After collection of tumor areas from brain sections by laser capture dissection microscopy and subsequent trypsin treatment, a total of 453 colony-forming phages was rescued of which 192 clones were randomly picked and analysed for full length nanobody expression and diversity. Dot blot analysis revealed that 95% of clones expressed nanobodies and restriction enzyme finger print analysis resulted in five different restriction patterns (not shown). As there is some chance of nanobody clones with similar restriction patterns being different on the nucleotide level, we arbitrarily chose to analyze from each group the 30% of clones with highest nanobody expression levels.

### Immunohistochemistry

**Figure 2:**
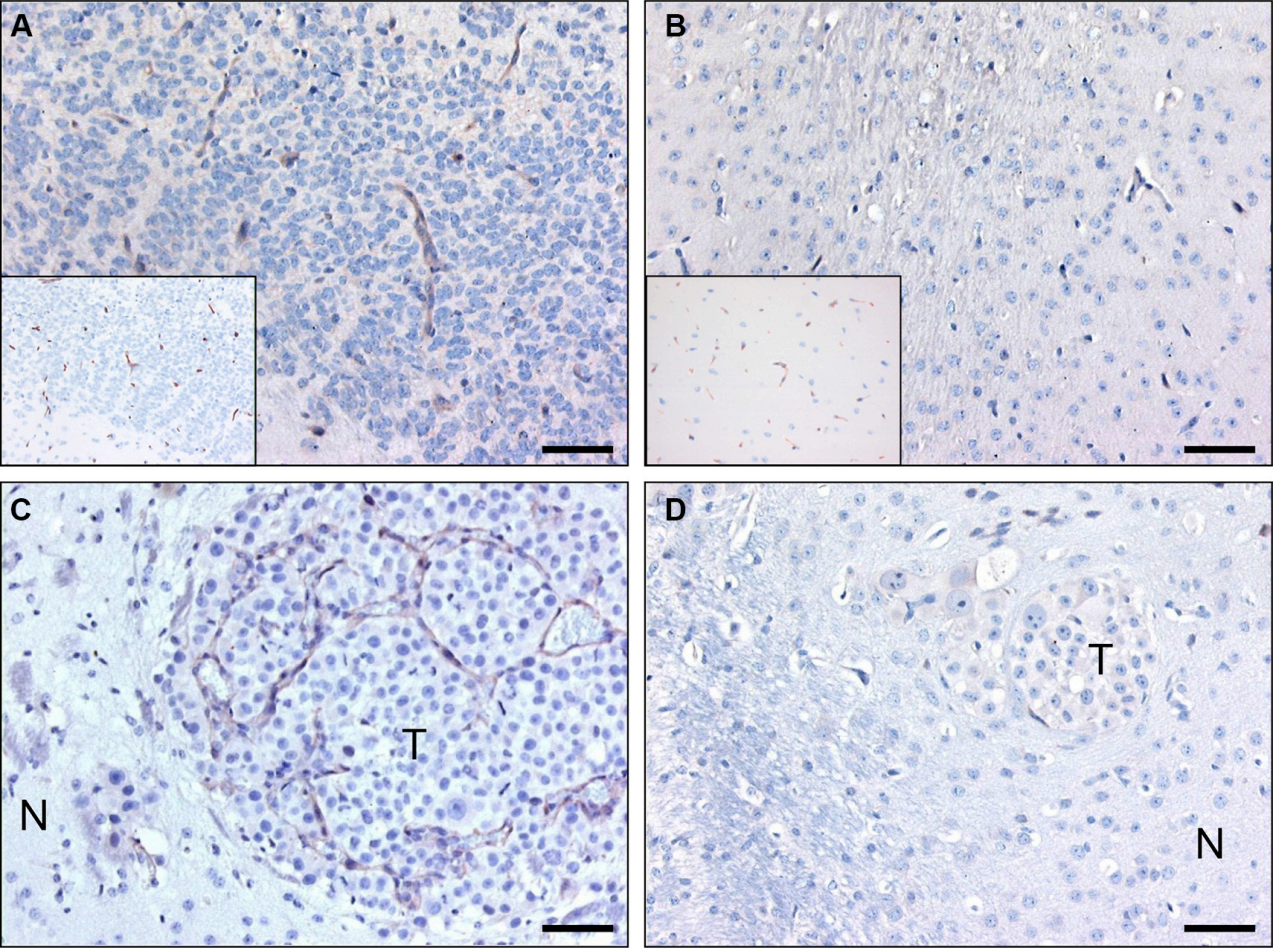
C-C7 recognizes tumor vessels in glioma xenografts and brain metastasis Immunohistochemical analysis of nanobody C-C7 in a diffuse-invasive part of cerebral E98 xenografts (**A**), normal mouse brain parenchyma (**B**), a representative brain metastasis of Mel57-VEGF_165_ melanoma (**C**) and a Mel57-VEGF-A165 xenograft after treatment with the VEGFR2 inhibitor vandetanib [42] (**D**). C-C7 recognizes subsets of tumor vessels in cerebral E98 lesions, while normal mouse brain vessels are negative. Insets in A and B show CD34 immunostainings of serial sections. Note that inhibition of VEGFR2 activity in panel D results in loss of C-C7 reactivity. N = normal, T = tumor. Bars correspond to 50 μm.

Immunohistochemical stainings were performed on sections of intracerebral E98 xenografts to select for nanobodies that specifically recognize tumor vessels. Because interpretation of staining of delicate capillaries requires optimal morphology, we chose to perform immunostainings on sections of FFPE-tissue blocks instead of cryosections, and accepted that potentially interesting nanobodies (recognizing conformational epitopes that are disrupted during formalin fixation) could be lost during analyses. Positive staining of blood vessels was observed with 27 of the 39 analyzed nanobodies of which 10 were not tumor-specific. The remaining 17 nanobodies stained subsets of tumor vessels. Of these, nanobody C-C7 showed most prominent staining of tumor vasculature in E98 xenografts and was analyzed in more detail. C-C7 stained both small and medium-sized tumor vessels in the diffuse infiltrative component of E98 xenografts [39, 40] (Figure 2A). Interestingly, not all tumor associated vessels were recognized (compare the C-C7 staining pattern with the anti-CD34 staining of the serial sections in the inset in Figure 2A). Nanobody C-C7 did not stain normal vasculature in mouse brain (Figure 2B). The observation that C-C7 stained only a subpopulation of tumor vessels prompted us to further characterize C-C7 by immunohistochemistry in other mouse tumor models. Nanobody C-C7 showed delicate staining of the vasculature in colorectal cancer xenografts (Supplementary Figure S1). Interestingly, blood vessels in angiogenic brain metastases of the human melanoma cell line Mel57-VEGF_165_ [41] were homogeneously positive (see representative example in Figure 2C) while staining was absent in Mel57-VEGF_165_ tumors after treatment with the VEGFR2 inhibitor vandetanib [42] (Figure 2D). This suggests that C-C7 recognizes VEGF-activated endothelial cells.

**Figure 3:**
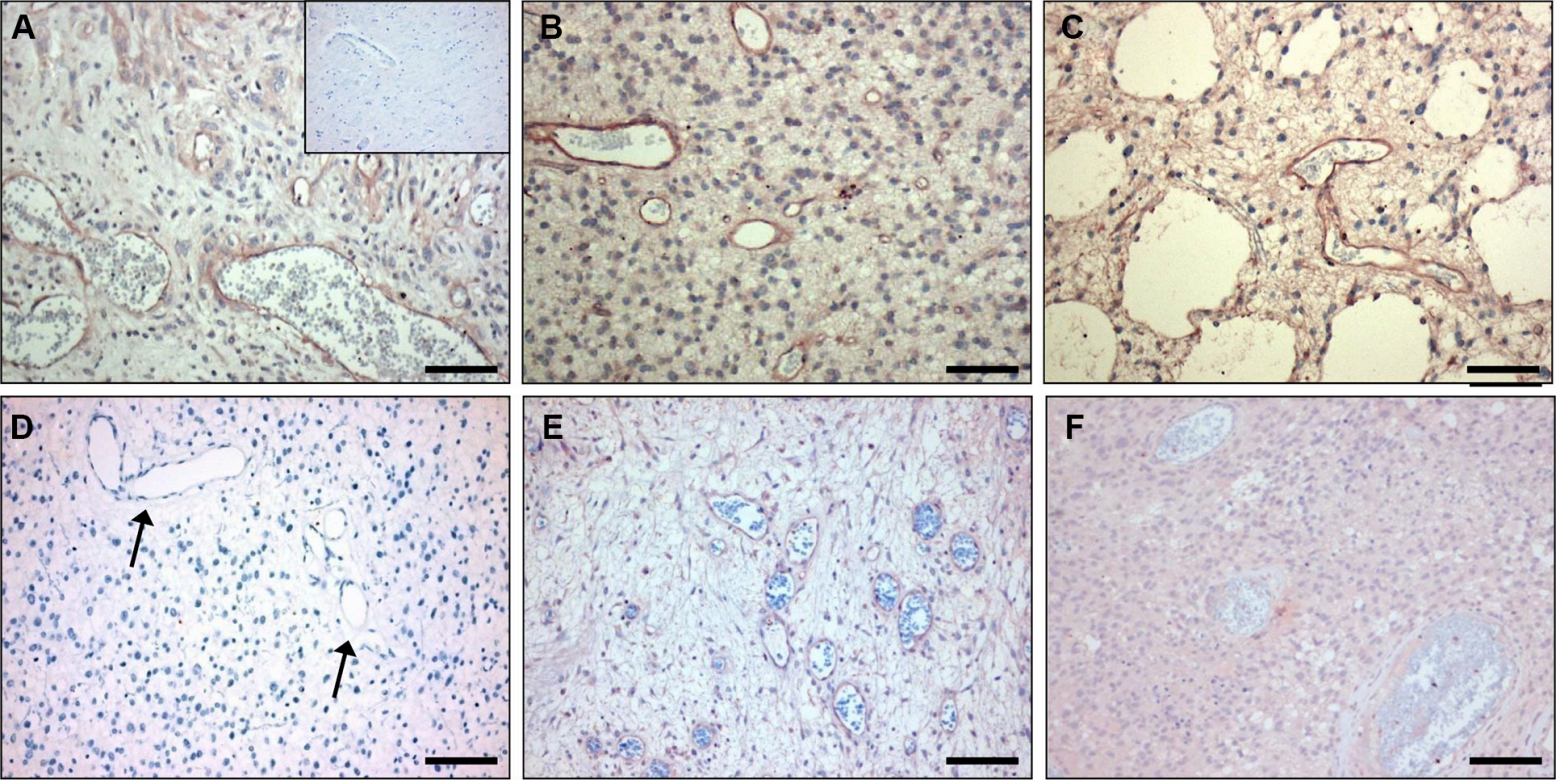
C-C7 recognizes tumor vessels in human glioblastoma multiforme Subsets of vessels in human glioma are strongly positive for C-C7 (**A**–**C**, **E**). Panels E and F represent a paired primary and recurrent tumor from a patient before (**E**) and after (**F**) treatment with bevacizumab and temozolomide. Note the absence of C-C7 reactivity on vasculature a low grade glioma (**D**) (arrow). The inset in panel A illustrates absence of C-C7 reactivity in the vasculature of normal brain. Bars in A-C correspond to 50 μm, in panels D–F to 100 μm.

To investigate whether nanobody C-C7 might have clinical relevance, we performed immunostainings on 9 clinical glioblastoma samples as well as normal human brain tissue. Subpopulations of tumor vessels in most glioma tissues stained positive for C-C7 (see representative examples in Figure 3A–3C and 3E) while C-C7 did not stain vasculature in normal human brain (inset in Figure 3A). In this set only one grade II glioma did not show any immunoreactivity towards C-C7 (Figure 3D). Of interest, paired glioma samples collected before and after avastin treatment [21], showed that vascular expression of the C-C7 ligand was absent after anti-angiogenic treatment (compare Figure 3E and 3F).

**Figure 4:**
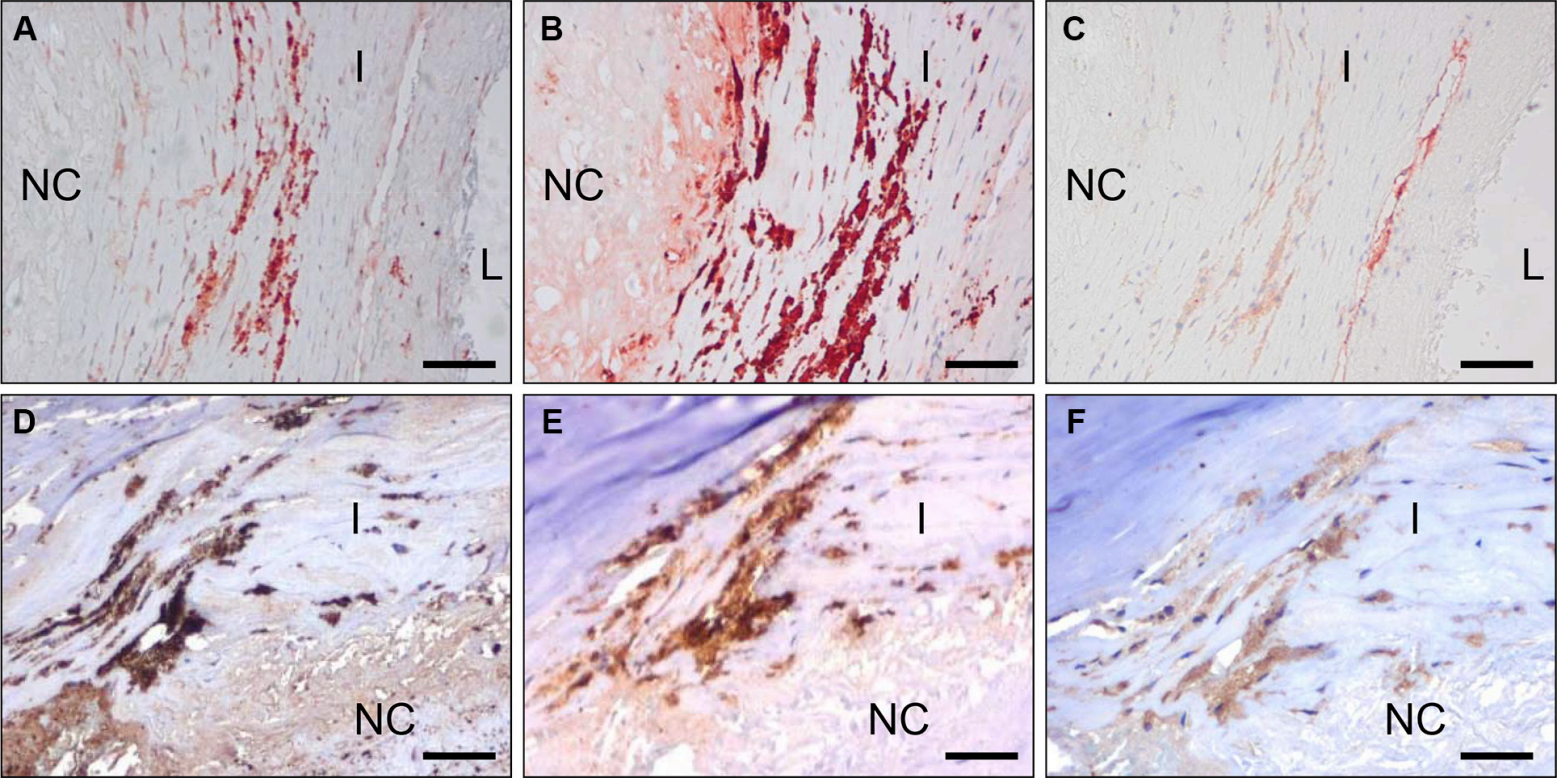
C-C7 recognizes blood vessels and macrophages in human atherosclerosis Immunostainings of surgical samples of atherosclerotic lesions from carotid arteries with CC7 (**A**, **D**) the macrophage marker CD68 (**B**, **E**) and the endothelial marker CD31 (**C**) and anti-dynactin-1 p150Glued (F, the anti-dynactin antibody was implemented in thee staining based on identification of dynactin as a binding partner of C-C7). (A–C) and (D–F) represent serial sections from two different lesions. Note the similarity in staining profiles for C-C7 and dynactin P150^Glued^ (D and F, respectively). Tissues in A–C were stained with AEC, tissues in (D–F) with DAB. NC = nectrotic core, I = intima, L = lumen. Bars correspond to 100 μM in panels (A–C) and to 50 μm in panels (D–F).

Vessel activation is not exclusive for cancers but also occurs in a number of other pathologies, e.g. atherosclerosis. Also in atherosclerotic lesions obtained from human carotid artery we observed CC7 immunostaining of endothelial cells, but also prominent staining of macrophages (compare C-C7 stainings in Figure 4A, 4D to CD68 stainings in Figure 4B, 4E and the CD31 staining in Figure 4C). Staining with anti-dynactin-1-p150^Glued^ (Figure 4F, see later for identification of dynactin-1-p150^Glued^ as the C-C7 target) showed clear colocalization with C-C7 (compare 4E and 4F).

**Figure 5:**
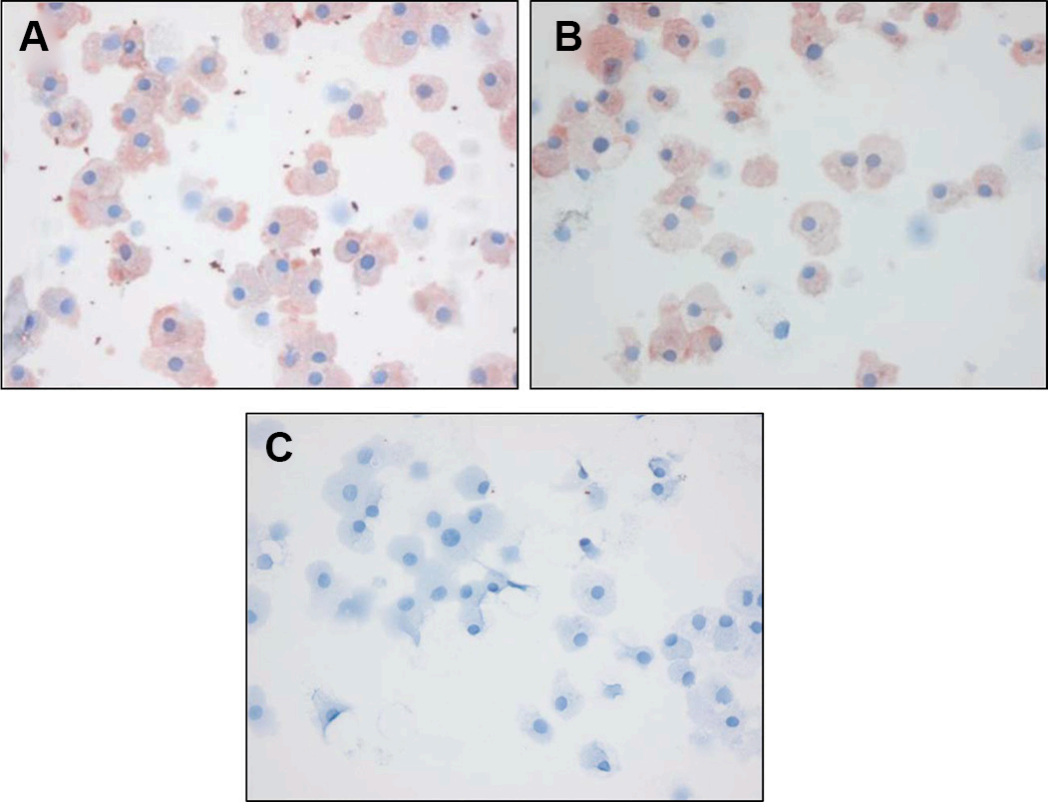
C-C7 recognizes dynactin-1 epitopes on macrophages Immunostainings with C-C7, anti-dynactin-p150^Glued^ antibody and an irrelevant control nanobody A9 on differentiated HoxB8 mouse macrophages. Note the similarity in staining profiles of C-C7 and dynactin-1 -p150Glued stainings.

To further investigate whether macrophage staining could be reproduced, we stained *in vitro* differentiated mouse HoxB8 macrophages with C-C7 and anti-dynactin-1-p150^Glued^. Both antibodies showed similar staining profiles. Control VHH A9 did not stain these cells (Figure 5).

### C-C7 can be used for targeting of cancer and atherosclerosis

**Figure 6:**
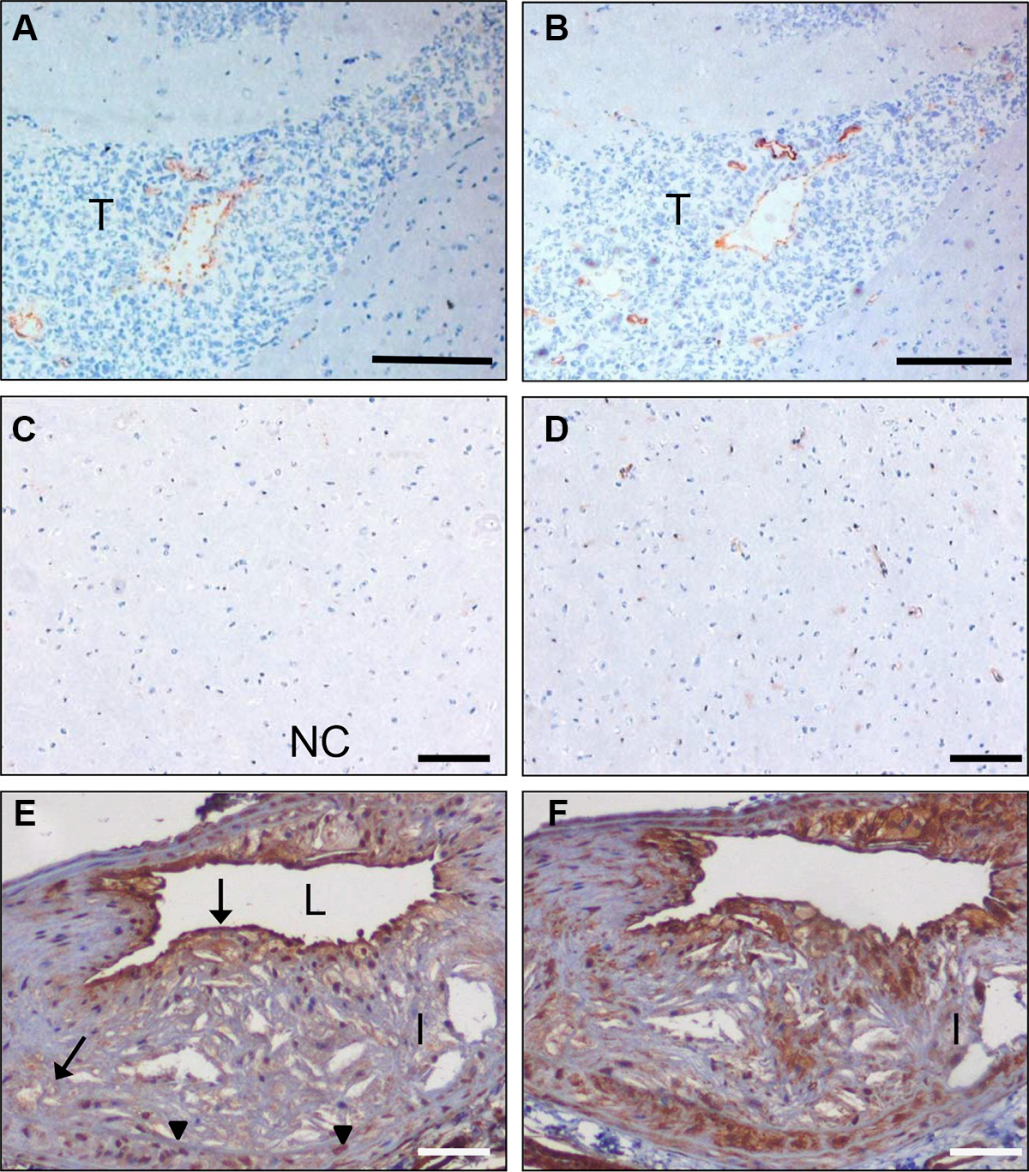
*In vivo* targeting of glioma xenografts and atherosclerotic lesions by C-C7-displaying M13 phages Monoclonal C-C7 phages were injected intravenously in mice carrying orthotopic E98 xenografts and phage distribution was analyzed by M13-p8 immunostaining after cardiac perfusion (**A**). A serial section was stained with CD34 to highlight vasculature (**B**). Phages displaying C-C7 home to a subpopulation of tumor vessels in E98 xenografts. Phages do not accumulate in normal vasculature in non-affected brain parts, as illustrated by M13-p8 (**C**) and CD34 (**D**) immunostainings. (**E**) M13-p8 immunostaining of atherosclerotic lesions of LDLR^−/−^ ApoB^100/100^ mice after an *in vivo* biodistribution experiment. Note that C-C7-phages home to luminal endothelium, intraplaque neovasculature (arrows) and macrophages (arrowheads). The M13-p8 immunostaining colocalizes with anti-Dynactin-1 immunostaining (**F**). T = tumor, I = intima, L = lumen. Bars in panels A, B correspond to 100 μm, in panels C-F: 50 μm

C-C7 was isolated from an *in vivo* biopanning experiment after intravenous injection, suggesting that it recognizes epitopes that are accessible to circulating nanobodies. To confirm the ability of C-C7 to target tumor vasculature, we injected monoclonal C-C7-phages in mice carrying orthotopic E98 xenografts, using the *in vivo* biopanning protocol which was used for its selection. C-C7-phages accumulated in subsets of vessels in E98 xenografts, but not in normal brain blood vessels (compare the phage localization in Figure 6A and 6C with the anti-CD34 staining of serial sections in Figure 6B and 6D). Control phages did not show specific tumor vessel localization, although some aspecific extravasation from leaky vessels was detected (data not shown). Similar experiments in LDLR^−/−^ ApoB^100/100^ mice carrying atherosclerotic lesions showed prominent homing of C-C7-phages to luminal endothelium, intraplaque neovasculature and macrophages (Figure 6E). C-C7-phages colocalized with dynactin-1-p150^Glued^ as shown by immunostainings (compare panels 6E and 6F).

### C-C7 recognizes the carboxyterminal domain of dynactin-1-p150^Glued^

**Figure 7:**
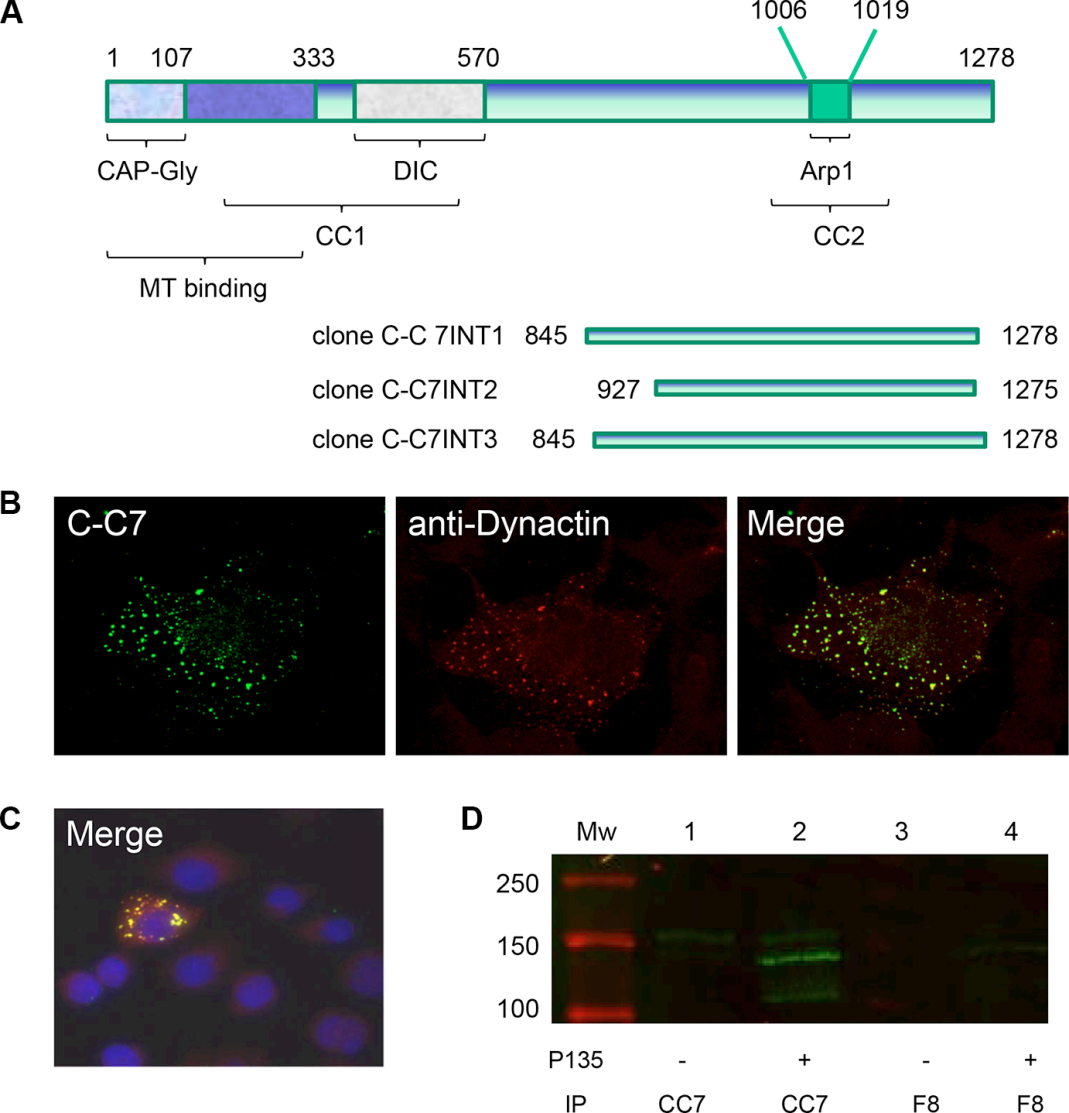
Dynactin-1-p150^Glued^ is identified as a C-C7 binding partner by a yeast-2-hybrid screen Structural domains of dynactin-1-p150^Glued^ and p150 domains identified by yeast-2-hybrid screens as C-C7 interactants (**A**). The aminoterminal domain contains a CAP-Gly domain and a coiled-coil domain which are responsible for microtubule (MT) binding and dynein binding (DIC = dynein intermediate chain). A second coiled-coil domain (CC2), encompassing a binding site for Arp1, is present in the carboxyterminal part of the protein and mediates binding to membrane components. Confocal microscopy showed colocalization of C-C7 (green) and commercial anti-dynactin-1-p150^Glued^ (red) in COS-1 cells, transfected with the recombinant carboxyterminal domain of dynactin-1-p150^Glued^ (**B**). Non-transfected cells did not stain with both antibodies (**C**), see negative DAPI-stained cells surrounding one transfected cell in this panel). C-C7 immunoprecipitates from extracts from CHO-s cells transfected with recombinant p135^Glued^ were analyzed on western blot using commercial anti-dynactin-1-p150^Glued^ as indicated (**D**). Note that p135 is readily precipitated by C-C7 (lane 2) but not by an irrelevant nanobody F8 (lane 4).

Since endothelial proteins are greatly underrepresented in tumor extracts, we argued that a proteomic approach to identify the C-C7 ligand would be unlikely to succeed. Therefore we decided to use nanobody C-C7 as bait protein in a Y2H screen against a large yeast expression library, using histidine (*HIS3*), adenine and β-galactosidase (*LacZ*) as reporter genes [43]. Three independent clones were identified from these screens which represented the carboxyterminal part of the p150^Glued^ subunit of dynactin-1. Clones C-C7-INT1 and C-C7-INT3 were composed of the carboxyterminal 432 amino acids of p150^Glued^ (aa 808–1240) and included 150 nucleotides of the 3′-untranslated region, while clone C-C7-INT2 spanned aa 890–1238 of the p150^Glued^ sequence (Figure 7A). A comparison of bovine dynactin-1-p150^Glued^ with the human homologue revealed 96% sequence identity in this region (not shown).

To confirm the interaction between C-C7 and human dynactin-1-p150^Glued^, the region encompassing aa 816–1278 (human numbering) was RT-PCR-cloned from human glioblastoma tissue and transiently expressed in COS-1 cells. Confocal microscopy of transfected cells, co-stained with nanobody C-C7 (visualized using FITC) and anti-dynactin-1 p150Glued (visualized using TRITC) showed that both antibodies co-localized to cytoplasmic vesicular structures (Figure 7B). Non-transfected cells did not stain with both antibodies (see Figure 7C).

### C-C7 specifically precipitates dynactin-1-p150^Glued^ and recombinant dynactin-1-p135^Glued^

Because colocalization of C-C7 with commercial antibodies as shown in Figure 7B and 7C formally does not exclude indirect interactions with other proteins than p150^Glued^ in the same vesicular structures, we wanted to further validate binding via immunoprecipitation. Under the conditions of cell lysis used in our experiments, the majority of carboxyterminal p150^Glued^ 816–1278 protein ended up in the pellet fraction (data not shown). As this problem did not occur with the p135-isoform of p150^Glued^ [44] we performed immunoprecipitation on extracts of p135-overexpressing CHO-s cells. P135 differs from p150 only in the N-terminal region, and hence contains the binding sites for both C-C7 and the commercial antibody. Furthermore, this variant can be readily distinguished from endogenous p150^Glued^ based on molecular weight. Figure 7D shows effective precipitation of p135 by C-C7 but not by irrelevant nanobody F8. Also, small amounts of endogenous p150^Glued^ were precipitated. In summary, these results confirm binding of C-C7 to p150^Glued^ and indicate that it is not an artefact of the Y2H screen.

**Figure 8:**
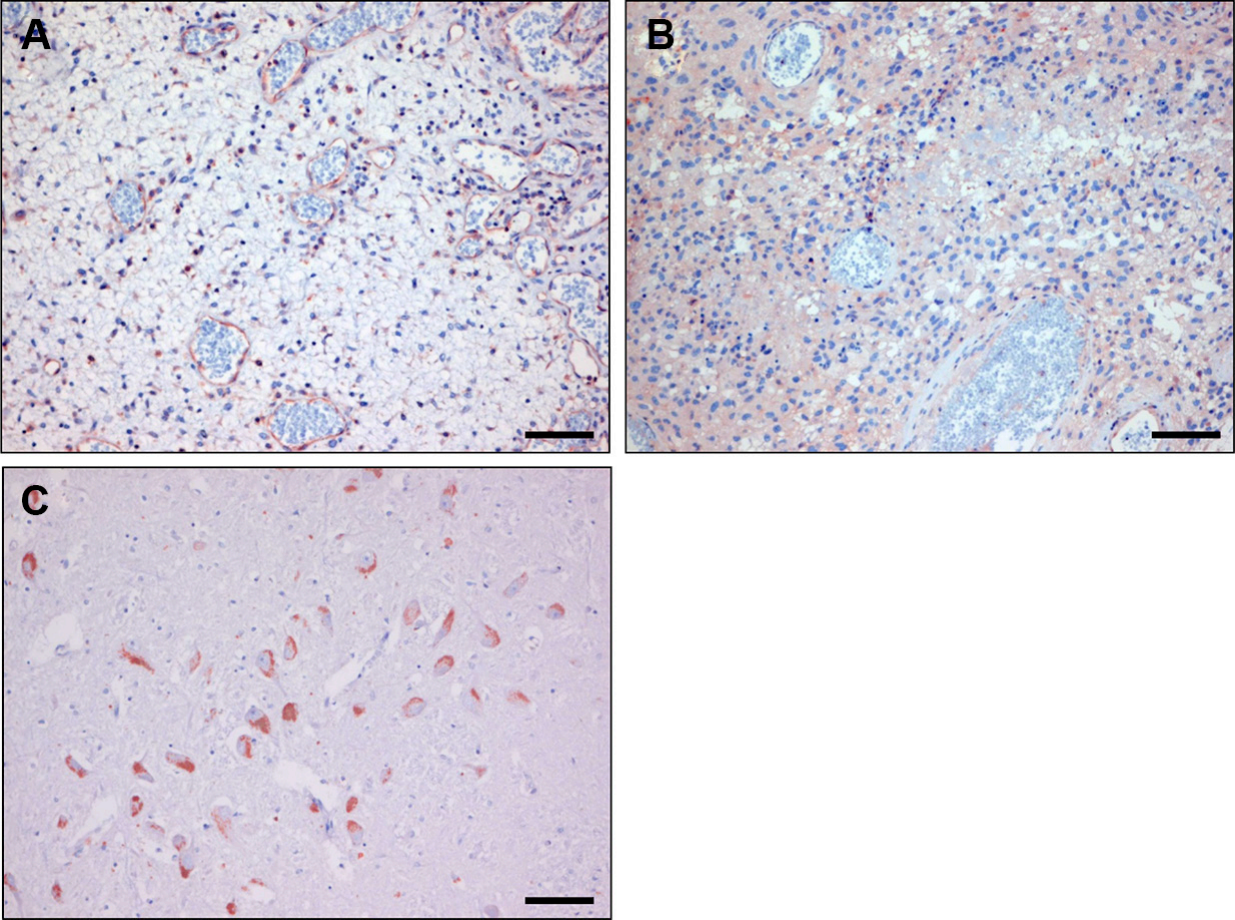
Immunostaining with anti-dynactin-1 -p150Glued of human glioblastoma before (**A**) and after bevacizumab treatment (**B**) Note the similarity with C-C7 immunostainings in Figure 3E and 3F. (Panel **C**) shows an anti-dynactin staining of normal brain, showing positive neurons, confirming literature data. Bars correspond to 100 μm.

To further validate that p150^Glued^ is expressed in tumor vasculature we performed immunostainings on clinical glioma samples with a commercially available rabbit anti-dynactin-1-p150^Glued^ antibody. In all tumors examined this antibody gave a similar staining profile as nanobody C-C7 (see Figure 8A and 8B respectively, note that these figures contain serial sections of the tissues presented in the C-C7 stainings in Figure 3E and 3F). Normal brain vasculature did not stain with anti-dynactin although positivity could be found on neurons, consistent with literature data [44] (Figure 8C).

## DISCUSSION

In human tumors the vasculature is in general highly heterogeneous with glioblastomas as a prime example [45, 46]. The diffuse infiltrative growth of human gliomas and the relatively undisturbed blood-brain barrier in these areas contribute to their poor response to surgery and radio- and chemotherapy [14, 47]. Gliomas do not adequately respond to anti-angiogenic therapies either [16, 21–23, 48] which is likely a consequence of angiogenesis-independent progression of diffusely infiltrating tumor cells. Therefore, a strategy of tumor-vessel targeting aiming at the induction of tumor infarctions is worth investigating. To this end (combinations of) targeting agents that specifically and selectively recognize tumor vasculature are needed.

Here we employed *in vivo* biopanning of a nanobody phage display library in the E98 orthotopic mouse model of diffuse glioma [19, 39]. This approach resulted in the isolation of a set of nanobodies of which 44% specifically recognized tumor blood vessels, indicating tumor-specific enrichment already during one round of biopanning. Presumably during the *in vivo* selection procedure, phages that bind to the surplus of common epitopes on quiescent endothelial cells in the circulation are competitively depleted, enriching for tumor-vessel specific binders in the tumor tissue. *In vivo* biopanning of phage display libraries therefore appears to be a relatively quick and efficient method to identify tumor targeting nanobodies.

In this study we concentrated on nanobody C-C7 because it proved to be the most suitable for immunostainings on formalin-fixed tissues. In line with the complexity of neovascularization and the resulting molecular heterogeneity of tumor blood vessels, we found that C-C7 stained only a subpopulation of blood vessels in glioma xenografts and human glioma, but also neovasculature and macrophages in atherosclerotic plaques. Interestingly, C-C7 stained VEGF-A165- activated endothelial cells in cerebral Mel57-VEGF-A165 xenografts, but not endothelial cells in blood vessels in these xenografts after treatment with the VEGFR2 inhibitor vandetanib [49]. In line, cerebral metastases of Mel57 xenografts that do not express VEGF-A and grow entirely via vessel co-option [41] also did not show vascular staining with C-C7 (not shown). This suggests that VEGF activation of endothelial cells is involved in expression of the C-C7 ligand, and this was corroborated in staining of paired surgical samples of a human glioblastoma before and after bevacizumab treatment. Of note, these data predict that tumor-vascular targeting with C-C7-like TVTAs should not be combined with anti-angiogenesis, as this would reduce the ‘activation-status’ of the vasculature and reduce targeting potential. Whatever the molecular underpinnings of C-C7 ligand expression, the approach of *in vivo* biopanning with nanobody-phage display libraries may yield proper candidates for further development of anti-vascular therapies, e.g. by generating nanobodies fused to truncated tissue factor, inducing tumor-vessel specific thrombosis [27, 50, 51]. A similar approach has been employed using phage libraries displaying peptides [52].

Using yeast-2-hybrid screens we identified dynactin-1-p150^Glued^ as the binding partner of C-C7 and we verified this interaction via co-immunostainings with nanobody C-C7 and commercial anti-dynactin-1- p150^Glued^ antibodies on tumor tissues, on atherosclerotic plaque tissues and p150^Glued^- transfected cells. Further validation came from immunoprecipitations of recombinantly expressed domains of p150^Glued^.

Dynactin-1-p150^Glued^ is involved in vesicular trafficking along microtubules, mitotic spindle assembly, cell migration and nuclear envelope breakdown during mitosis [53–55]. Co-immunostaining of COS-1 cells transiently overexpressing the carboxyterminal part of dynactin-1- p150^Glued^, with nanobody C-C7 and a commercial anti-dynactin-1- p150^Glued^ antibody showed a vesicular pattern, in agreement with a previous study on a dynactin-p150^Glued^ mutant that is defective in microtubule binding [56].

Since our biopanning protocol was designed to identify tumor-vessel associated proteins that are targetable via intravenous injection, the binding of nanobody C-C7 to the alleged intracellular protein p150^Glued^ was unexpected. Which mechanisms are involved in extracellular presentation of dynactin-1-p150^Glued^ making it accessible to intravenously administered phages, is unclear. However, a number of proteins have been shown to use non-conventional ways of trafficking in the cell [57]. Extracellular presentation of otherwise cytoplasmic or even nuclear proteins in a tumor context has been described before, examples being transglutaminase, fatty acid binding protein 3, and GRP78, although also for these targets the molecular basis for their extracellular localization is still enigmatic [52, 58–61]. A possible mechanism explaining extracellular presentation of p150^Glued^ involves exocytosis of dynactin-decorated vesicles via multivesicular endosomes [62, 63]. After exocytosis of these vesicles, fusion with activated endothelial cells may lead to extracellular p150^Glued^ presentation. This resembles the horizontal transmission of tumor targets via exosomes that has been proposed as an explanation for endothelial expression of tumor-derived EGFRvIII [64–66]. Of note, we also observed high expression of dynactin-1-p150^Glued^ in macrophages in atherosclerotic lesions. Based on our immunohistochemical analyses we conclude that dynactin-1-p150^Glued^ is not expressed at high levels in tumor cells. The actual source of dynactin-1-150^Glued^ may therefore be activated macrophages/microglial cells. These issues are currently under investigation.

In conclusion, *in vivo* biopanning of phage nanobody-display libraries in animal models of cancer in combination with Y2H technology represents a powerful platform to identify novel TVTAs and their binding ligands. We identified C-C7 as a targeting nanobody against dynactin-1-p150^Glued^ in a glioma xenograft model and in a model of atherosclerosis. It remains to be seen whether this nanobody can be used for therapeutic anti-vascular targeting purposes.

## MATERIALS AND METHODS

### Tumor models

All animal experiments were approved by the Animal Experiment Committee of the RadboudUMC. Orthotopic E98 glioma xenografts were established in 6–8 week old Balb/c nude mice by intracerebral injections of E98 tumor cell suspensions as described previously [18]. In this model mice characteristically start to display weight loss and neurological symptoms due to tumor growth 3–4 weeks after tumor implantation. When tumor-related symptoms were apparent, mice were used for *in vivo* biopanning.

### *In vivo* biopanning

Hundred μl of phosphate buffered saline (PBS) containing 10^12^ phages of a nanobody-displaying phage library (cloned from lymphocytes of non-immunized Llama glama in phagemid pHENIX-HIS-VSV [28]) was injected in the tail vein of nude mice, carrying cerebral E98 tumors (*n* = 2). Phages were allowed to circulate for 15 minutes. Mice were subsequently put under deep anaesthesia with a mixture of isoflurane/N_2_O and chests were opened. Cardiac perfusion was performed with 10 ml sterile 0.9% NaCl solution to remove unbound phages from the circulation. Brains were removed and snap frozen in liquid nitrogen. Sections of 4 μm were stained with anti-M13 p8 antibody (Abcam Limited, Cambridge, UK) to assess phage distribution. Using a laser dissection microscope (Leica AS LMD, Wetzlar, Germany) tumor areas were dissected from 10 μm brain sections. Phages were eluted from these sections by incubation with trypsin (10 mg/ml in PBS) for 30 min at room temperature. Following infection in 10 ml of log-phase *E. coli* TG1 culture, bacteria were seeded on 2xTY plates containing 100 mg/L ampicillin and 2% glucose. After overnight incubation, individual clones were picked and analyzed via colony-PCR for the presence of full-length nanobody insert using flanking primers M13rev (5′-TCA CAC AGG AAA CAG CTA TGA-3′) and Fedseq3 (5′-GTA ACG ATC TAA AGT TTT GTC G-3′). Subsequently, PCR products were digested with the 4-cutter restriction enzyme *Bst*NI (New England Biolabs, Ipswich, MA, USA) to analyze the diversity of the phage population. Small scale production of soluble nanobodies by independent clones was induced in log-phase TG1 cells by culturing at 30^°^C in 2 × TY medium, containing 100 mg/L ampicillin and 1 mmol/L isopropylthiogalactoside (IPTG, Serva, Heidelberg, Germany). Expression of nanobodies was verified by dot blot analysis of medium using anti-VSV antibodies, as previously described [28]. Based on combined data of full length PCR, *Bst*NI restriction digestion and dot blot analysis, expression of selected individual nanobodies was induced in 50 ml log-phase TG1 cells with 1 mM IPTG for 3 hours at 30^°^C. Nanobodies were isolated from the periplasm by osmotic lysis and purified by Ni-NTA sepharose (IBA Life Sciences, Gottingen, Germany) as described previously [28].

### Immunohistochemistry

Nanobodies were tested in immunohistochemical stainings on 4 μm sections of formalin-fixed paraffin-embedded (FFPE) tissues and tumor xenografts of different origin (intracerebral E98 xenografts; brain metastases of Mel57-VEGF_165_ melanoma after treatment with vandetanib or controls [49]; subcutaneous Mel57-VEGF_165_ xenografts and subcutaneous xenografts of the colon carcinoma cell lines C26 and C38 [67]). Human gliomas, classified by an experienced neuropathologist as grade II (*n* = 2) and grade IV (*n* = 7) gliomas, as well as normal brain tissue (*n* = 2) were retrieved from the archives of the departments of Pathology of RadboudUMC and of the Academic Medical Center. GBM patients provided written informed consent, and the study was conducted in accordance with the Declaration of Helsinki and was approved by the Academic Medical Center Institutional Review Board and the Dutch Central Committee on Research investigating Human Subjects (ISRCTN23008679). Use of the other archival tissues was in accordance with institutional guide lines.

Following deparaffinization, endogenous peroxidase activity was blocked by incubation with 3% H_2_O_2_. Antigen retrieval was performed by boiling in 10 mmol/L citrate buffer (pH 6.0). Sections were pre-incubated with normal goat serum to block non-specific binding sites, followed by overnight incubation with 3 μM nanobody in PBS/2%BSA at 4^°^C. Detection was performed by sequential incubations with rabbit anti-VSV-G antibody (for mouse tissues, Sigma-Aldrich Chemie B.V., Zwijndrecht, The Netherlands,) or anti-VSV-G P5D4 (for human tissues), biotinylated anti-rabbit antibody (Vector, Burlingame, CA) and avidin-biotin peroxidase complex (Vector). Peroxidase was visualized with 3-amino-9-ethylcarbazole (AEC, ScyTek, Utah, USA) with haematoxylin as counterstain. Tumor vessels in mouse xenograft tissues were visualized in serial sections with rat-anti-mouse CD34 antibody (Hycult, Uden, The Netherlands). Rabbit antibodies against dynactin-1-p150^Glued^ were from Abcam (Cambridge, UK).

### C-C7 in atherosclerosis

Carotid artery atherosclerotic tissue from patients at risk for ischemic stroke was obtained from routine surgical procedures at the department of Vascular Surgery of RadboudUMC (kindly provided by Dr. J. Pol). Directly after surgery tissues were processed to FFPE blocks. Four μm sections were deparrafinized and pretreated as described, and incubated with 3 μM C-C7 in normal antibody diluent (Immunologic, Duiven, The Netherlands). Then slides were incubated with anti-VSV-G P5D4, PolyHRP-anti-Ms/Rt/Rb (Immunologic, Duiven, The Netherlands), and peroxidase was visualized with bright 3′3Diaminobenzidine (DAB) (Immunologic, Duiven, The Netherlands).

Furthermore, specificity of C-C7 was assessed by staining on mouse macrophages, obtained by differentiating ER-HoxB8 myeloid precursor cells by 25 ng/ml GM-CSF on 8-well Lab-Tek Chamber Slide™ System (Nunc, Roskilde, Denmark) [68]. Cells were fixed with 2% paraformaldehyde for 10 min, and pre-incubated with normal goat serum. Then cells were incubated with 3 μM C-C7 or rabbit-anti-Dynactin-1-P150^Glued^ in PBS/2%BSA. Detection was performed as described above in immunohistochemistry.

In another set of experiments hypercholesterolemic (low-density lipoprotein receptor (LDLR)^−/−^ apolipoprotein B (ApoB)^100/100^) mice [69] were subjected to high-cholesterol diet (TD 88137, Harlan Laboratories, Indianapolis, IN, USA) for 8 weeks after which a peri-adventitial collar was placed around a carotid artery. Hundred μl PBS containing 10^12^ M13-phages displaying nanobody C-C7 or helper phages as control, was injected via the tail vein and allowed to circulate for 15 minutes after which a cardiac perfusion with 10 ml PBS was performed. Lesions from the carotid artery were harvested and processed to FFPE blocks, sections of which were stained with anti-M13 p8 or with anti-dynactin-pGlued^150^ antibody.

### Y2H screens

All cloning steps were performed using the Gateway System (Invitrogen, Carlsbad, CA). The cDNA encoding nanobody C-C7 (without pelB leader) was flanked by attB sites via PCR and cloned in vector pDONR201 before transfer into destination vector pBD-GAL4-CAM/DEST, generating a C-C7 fusion protein with the GAL4 DNA binding domain. After sequence-verification this vector was transfected into yeast strain PJ69 (A-mating type) using standard protocols [43]. Generation of a randomly primed bovine retina cDNA library in pAD-GAL4, transfected in PJ69 (α-mating type, 2 × 10^6^ clones) has been previously described [43]. The yeast strain containing pBD-GAL4-C-C7 was mated with this library and diploid cells in which C-C7-prey interactions led to functional GAL4 transcription factor, were selected based on histidine and adenine prototrophy and transactivation of ß-galactosidase activity. Production of ß-galactosidase by activation of the *LacZ* reporter gene was detected by a filter-lift assay [43]. From positive yeast clones, pAD-GAL4 library expression plasmids were rescued and amplified in *E. coli*. Plasmids were sequenced using flanking forward and reverse primers. Sequences were blasted (http://www.ncbi.nlm.nih.gov/blast/Blast.cgi) and aligned with multalin software (http://bioinfo.genotoul.fr/multalin/multalin.html). All sequenced clones contained intact reading frames in fusion with the GAL4-activation domain.

### Verification of the C-C7-dynactin-1 interaction

The carboxyterminal part of dynactin-1-p150^Glued^ was RT-PCR-cloned from human glioblastoma cDNA using primers attB1-dyn 2532 (5′-AAA AAG CAG GCT TCA CCA TGG CAG CTG CTG CTG CC-3′, sense) and attB2-dyn 3837 (AGA AAG CTG GGT GTT AGG AGA TGA GGC GAC TGT G-3′, antisense). AttB1 and attB2 sites were extended in a second PCR and products were cloned via pDONR201 in p3xflg-CMV (Invitrogen, Carlsbad, CA, USA) to generate p3xflg-CMV-dyn 2532–3837. Plasmids were transfected into COS1 cells in 8-well glass slides (Lab-Tek Chamber Slide™ System, Nunc, Roskilde, Denmark). Forty-eight hours later, cells were fixed with ice-cold acetone and incubated with nanobody C-C7 for 1 hr, followed by sequential incubations with mouse anti-VSV and goat-anti-mouse FITC (Invitrogen, Carlsbad, CA, USA). After washing, slides were stained with rabbit-anti-dynactin-1 which was detected with donkey-anti-rabbit TRITC (Invitrogen, Carlsbad, CA, USA). Images were processed on a Leica Discovery Fluorescence Microscope and a Leica confocal microscope.

### Immunoprecipitation studies

In another set of experiments, the p135 variant of dynactin [44] was obtained via RT-PCR from glioblastoma RNA using primers P135fw-*Cla*I (5′-CATCGATACCATGATGAGACAGGCACC-3′) and P135rev-*Not*I (5′ CAGCGGCCGCTTAGGAGATGA GGCGACTG-3′) and digested and cloned in *Cla*I-*Not*I digested pIRESneo (Clontech, Mountain View, CA). Suspension cultures of Chinese Hamster Ovary cells (CHO-s) in SFM4CHO medium with L-glutamin (ThermoFisher, Landsmeer, The Netherlands) were transfected with pIRES-Dynactin-p135 using the Amaxa Nucleofactor kit according to the manufacturer's instructions (Lonza, Cologne, Germany). Cells were harvested 48 hours later and lysates prepared in lysis buffer [50 mM Tris-HCl, pH 7.4, 1% Triton-X100, 150 mM NaCl and protease inhibitors (Complete^TM^ protease inhibitor cocktail, Roche, Mannheim, Germany)] with untransfected CHO-s cells serving as control. Lysates were clarified at 13,000 g for 10 min at 4^°^C. Dynactin-p135 expression was verified on western blot using rabbit-anti-Dynactin (AbCam Cambridge, UK).

For immunoprecipitations, periplasmic extracts of His-tagged C-C7 or irrelevant control nanobody F8 were diluted five times in IPP_500_ (50 mM phosphate buffer pH 8.0, 500 mM NaCl) and incubated with Ni-NTA sepharose for 2 hours at 4°C. After washing three times with IPP_500_ and three times with IPP_150_ (50 mM phosphate buffer, pH 8.0, 150 mM NaCl) beads with immobilized C-C7 were incubated with cleared cell lysates for 2 hours at RT. Beads were washed two times with IPP_150_ incubated with 5 mM imidazole in IPP_150_ for 10 min at RT, boiled for 5 minutes in reducing sample buffer and loaded on a 7% SDS-PAGE gel for Western blot analysis with rabbit anti-dynactin and goat anti-rabbit IRDye_800_ (LI-COR, Lincoln, NE, USA) using the Odyssey infrared imaging system (LI-COR, Lincoln, NE, USA).

## SUPPLEMENTARY MATERIALS FIGURES


