# Supplementary material for: In vivo phage display screening for tumor vascular targets in glioblastoma identifies a llama nanobody against dynactin-1-p150Glued
Source: Oncotarget. 2016 Sep 26;7(44):71594–607. doi: 10.18632/oncotarget.12261 (PMC5342104; doi:10.18632/oncotarget.12261)
Supplement: Supplementary file 1 [file oncotarget-07-71594-s001.pdf]

In vivo phage display screening for tumor vascular targets in glioblastoma identifies a llama nanobody against dynactin-1-p150^{Glued}

Supplementary Materials

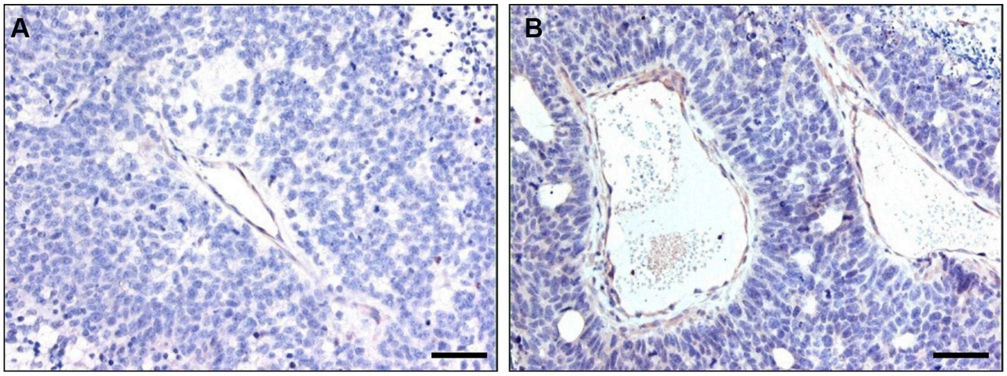

Supplementary Figure S1: C-C7 recognizes tumor vessels in colon carcinoma xenografts. Imunohistochemical analysis shows that C-C7 recognizes vasculature in subcutaneous colorectal cancer xenografts C26 (**A**) and C38 (**B**). Bars correspond to 50 µm.
